# Water Quality Modelling for River Activities Management: Example from a Low- and Middle-Income Country

**DOI:** 10.5696/2156-9614-10.28.201207

**Published:** 2020-12-02

**Authors:** Izni Zahidi, Geoffrey Wilson, Katherine Brown, Felix Ku Kok Hou

**Affiliations:** 1 Civil Engineering Discipline, School of Engineering, Monash University Malaysia, Selangor, Malaysia; 2 Sustainable Development and Climate Change, Asian Development Bank, Philippines; 3 Water Resources Department, DHI Water and Environment, Kuala Lumpur, Malaysia

**Keywords:** water quality, modelling, sand mining, aquaculture, Malaysia

## Abstract

**Background.:**

Rivers are susceptible to pollution and water pollution is a growing problem in low- and middle-income countries (LMIC) with rapid development and minimal environmental protections. There are universal pollutant threshold values, but they are not directly linked to river activities such as sand mining and aquaculture. Water quality modelling can support assessments of river pollution and provide information on this important environmental issue.

**Objectives.:**

The objective of the present study was to demonstrate water quality modelling methodology in reviewing existing policies for Malaysian river catchments based on an example case study.

**Methods.:**

The MIKE 11 software developed by the Danish Hydraulic Institute was used to model the main pollutant point sources within the study area - sand mining and aquaculture. Water quality data were obtained for six river stations from 2000 to 2015. All sand mining and aquaculture locations and approximate production capacities were quantified by ground survey. Modelling of the sand washing effluents was undertaken with the advection-dispersion module due to the nature of the fine sediment. Modelling of the fates of aquaculture deposits required both advection-dispersion and Danish Hydraulic Institute ECO Lab modules to simulate the detailed interactions between water quality determinants.

**Results.:**

According to the Malaysian standard, biochemical oxygen command (BOD) and ammonium (NH_4_) parameters fell under Class IV at most of the river reaches, while the dissolved oxygen (DO) parameter varied between Classes II to IV. Total suspended solids (TSS) fell within Classes IV to V along the mid river reaches of the catchment.

**Discussion.:**

Comparison between corresponding constituents and locations showed that the water quality model reproduced the long-term duration exceedance for the main body of the curves. However, the water quality model underestimated the infrequent high concentration observations. A standard effluent disposal was proposed for the development of legislation and regulations by authorities in the district that could be replicated for other similar catchments.

**Conclusions.:**

Modelling pollutants enables observation of trends over the years and the percentage of time a certain class is exceeded for each individual pollutant. The catchment did not meet Class II requirements and may not be able to reach Class I without extensive improvements in the quality and reducing the quantity of both point and non-point effluent sources within the catchment.

**Competing Interests.:**

The authors declare no competing financial interests.

## Introduction

Rivers are critical freshwater resources, but also among the most vulnerable. River water quality is crucial to ensuring livelihoods, food production, energy generation, industry and environmental support. Rivers are exposed to pollution which is a key challenge requiring intervention from various stakeholders. Rapid population growth also means an inevitable increase in water demand. Surface water from rivers and streams are sensitive to pollution as they are major sources of fresh water. Water pollution is especially common in low- and middle-income countries (LMIC) where development is rapid and environmental considerations are minimal.

Water quality studies are essential for river activities management and policy making.^1–3^ A framework should be formulated to regulate and control the main sources of pollution. In order to address pollution sources, the existing river condition and pollution sources must first be identified. Land use changes such as increasing sand mining and agriculture activities almost always leads to a decline in water quality and therefore, there is a need for strategic planning to mitigate impacts to achieve the desired water quality objectives.^4,5^ An important part of strategic planning requires data collection on existing water quality conditions at a local scale.

An overall indicator used to classify the water quality of a watercourse is the water quality index (WQI), a set of weighted and averaged mathematical equations to summarize the water quality of a river that includes total suspended sediment (TSS), dissolved oxygen (DO), biochemical oxygen demand (BOD) and ammonia (NH_4_).^6,7^ A sufficient DO concentration indicates a healthy ecosystem and low concentrations indicate organic pollution. Biochemical oxygen demand is a measure of oxygen demand released in water caused by decaying organic matter. Dissolved oxygen and BOD have an inverse correlation. Ammonia consists of nitrogen which is a key nutrient for algal growth. It requires only a small amount of nitrogen, therefore levels in excess of 0.1 mg indicate contamination and pose a threat to the environment.

In Malaysia, approximately 150 major river basins supply water for irrigation and domestic uses with very little regulation. Over the years, there have been a number of studies assessing water quality in the local river catchments that point to the necessity for specific frameworks to monitor these activities.^8–11^ Malaysia uses the National Water Quality Standards (NWQS) to classify river water quality based on individual pollutants. Looking into the effects of sand mining on the rural Kelantan River, Yen and Rohasliney found that TSS, turbidity and nitrate contents exceeded the NWQS and Al-Badaii *et al.* assessed the water quality of the Semenyih River in Selangor during both the dry and rainy seasons in 2012.^8,9^ The authors concluded that both rivers can be used for irrigation, but they required extensive treatment for domestic uses. However, due to the single point in time measurement nature of these studies, the water quality target is constant for all river branches/tributaries for all uses. Specific targets for individual river branches corresponding to specific intended uses would be beneficial.

**Abbreviations**
*BMP*: Best management practices
*BOD*: Biochemical oxygen demand
*DO*: Dissolved oxygen
*DOE*: Malaysian Department of Environment
*NWQS*: National Water Quality Standards
*SS*: Suspended solids
*TSS*: Total suspended solids
*WQJ*: Water quality index

While conventional field measurements are sufficient for many water quality studies, more applications have been shown to benefit from water quality modelling. Modelling pollutants enables investigations of trends over the years and the percentage of time a certain class is exceeded for each individual pollutant. Mathematical models have been used for the same purposes, but their application in risk assessment and management is limited due to the difficulty in preparing the input data, particularly the pollutant loads and explaining results.^11^ It is important to see water quality modelling as a supporting tool for water quality assessment that would provide tangible information to enhance water quality monitoring and river activities management.

Othman *et al*. for example, used modelling to map the 12-km urban Penchala River in Malaysia which suffered from residential and industrial waste discharges.^11^ Othman and Elamin extended the methodology to a different urban river in Klang using only two parameters: BOD and DO.^12^ A similar type of map was produced for the Klang River to support informed decision making. Hashim and Teo carried out a similar approach of water quality modelling for the Kuyoh River catchment in the state of Selangor for the same parameters at three limited points.^10^ Aminu *et al.* modelled the Bertam River in the Cameron Highlands in the state of Pahang which is negatively affected from tourism-related activities, and calculated WQI at specific sampling locations.^13^ The data were subsequently interpolated to predict the values along other reaches.

All of these studies proposed a number of recommendations to improve river water quality. However, there was no further analysis of the framework or attempt to formulate one. They are also outdated as land use changes in some areas can be rapid and there is a need to carry out continuous monitoring and simulation to investigate trends as well as to help formulate the framework required to ensure that pollution is controlled in the long term. Liang *et al*. used the same model as the one proposed in this project as a tool to evaluate water quality management plans for the Beijing River in China.^14^ Using a different model, Hutchins and Bowes modelled the impacts on abstraction location changes on the water quality downstream of the River Thames.^15^ In both cases, water quality modelling has been successfully used to simulate various scenarios varying from worst case to mitigation options to determine if a plan can achieve the project objectives.

Therefore, the objective of the present study is to propose a water quality modelling methodology and review existing policies for updating the effluent disposal standards for Malaysian river catchments based on an example case study. This study can serve as the foundation for legislation and regulations by authorities for two common river activities in Malaysia - sand mining and aquaculture. At the moment, different states in Malaysia adopt general standards only and updated policies are needed.

## Methods

Due to the location sensitivity, the study area is a non-disclosed river channel in a Malaysian state with a catchment area of 1300 km^2^. The river passes through the city and several industrial estates prior to discharging into the sea. The upper part of the catchment is mountainous while the remainder is comprised of hilly undulating land with some swamps in the lower reaches of the river. A large portion of the catchment has been converted from forest to agricultural, industrial, commercial and residential uses. This study area was selected due its booming sand mining and aquaculture activities which have the potential to negatively impact river water quality. Water quality data were obtained from the Malaysian Department of Environment (DOE). All sand mining and aquaculture locations and approximate production capacities have been quantified and supplemented by ground survey.

The modelling process that has been applied to this work aims to model pollution sources at the local site to describe temporal and spatial variation of physical processes to inform the assessment of management strategies and pollution. The software MIKE 11 developed by the Danish Hydraulic Institute was selected for this project as its customizable ECO Lab water quality module has the capability to simulate detailed interactions between water quality determinants.^16^

The main pollutant point sources within the study area have been identified as sand washing activities at the sand mining sites and aquaculture farms. Sediment loadings resulting from sand washing activities together with pollutant loadings from the aquaculture farms have been estimated based on measurements collected from the site reconnaissance surveys conducted during the course of the study. Modelling of the sand washing effluents was undertaken solely with the advection-dispersion module of the MIKE software due to the nature of the sediment which mainly consists of fine sediment. The model has been calibrated where possible to observed measurements. Modelling of the fates of aquaculture deposits required both the advection-dispersion and ECO Lab modules. A base ECO Lab template has been used to describe the oxygen and nitrogen processes. The ECO Lab module setup includes the variables, processes and constants as set out in Table 1.

**Table 1:** Variables, Forcings and Processes in MIKE ECO Lab Modules

**Variables and forcings**	DO
	Temperature
	NH_4_
	Nitrate
	BOD
	Salinity
	Water depth
	Flow velocity
	Slope
**Processes**	BOD degradation
	Reaeration
	Photosynthesis in water column
	Radiation into water
	Radiation out of water
	Sediment oxygen demand
	Sedimentation of BOD
	BOD re-suspension
	Ammonification
	Nitrification
	N uptake plants
	N uptake bacteria
	Oxygen consumption nitrification
	Denitrification

Abbreviation: N, nitrogen.

Water quality calibration has been performed by comparing model results against long-term measurement samples (year 2000–2015) collected at the sampling stations within the study area: Station 01 (downstream), Station 02, Station 03, Station 04, Station 05 and Station 06 (upstream). The gauged water quality data for the period February 2000 to November 2015 are available for the six sampling stations within the catchment. These water quality recordings include six constituents, including DO (mg/l and %), BOD (mg/l), chemical oxygen demand (mg/l), TSS (mg/l), pH and NH_4_ (mg/l).

An overall WQI was derived using the general WQI formula in Equation 1.



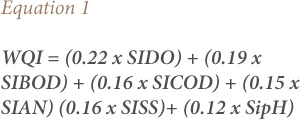
where,

SIDO = Sub-index DO (% saturation)
SIBOD = Sub-index BOD
SICOD = Sub-index chemical oxygen demand
SIAN = Sub-index NH3-N
SISS = Sub-index suspended solids (SS)
SipH = Sub-index pH
0 ≤ WQI ≤ 100

Subsequently, an overall water quality class has been assigned for each sample instance based on DOE classification as tabulated in Table 2.^17^

**Table 2:** Water Quality Classification^17^

**Class**	**Uses**
I	Conservation of the natural environment Water supply I - practically no treatment is necessary (except disinfection by boiling only) Fishery I - very sensitive aquatic species
II	Water supply II - conventional treatment required Fishery II - sensitive aquatic species Recreational use with body contact
III	Water supply III - extensive treatment required Fishery III - common, of economic value and tolerant species
IV	Irrigation
V	None of the above

A summary of the observed water quality class categorization for each sampling station is tabulated in Table 3. The class at the sampling station is not constant as it depends on many aspects, primarily actual upstream loading and actual flow rate. This mirrors the results of the water quality simulations showing that the pollutant concentrations, and therefore the class vary; in some cases between Class I and Class IV (Station 06).

**Table 3:** Summary of Observed Overall WQI as a Percentage of All Observations

**Sampling station (downstream - upstream**	**Observed WQI class**			

	**I**	**II**	**III**	**IV**
Station 01	0%	71%	29%	0%
Station 02	0%	28%	72%	0%
Station 03	0%	23%	75%	2%
Station 04	0%	19%	76%	5%
Station 05	0%	10%	90%	0%
Station 06	1%	24%	74%	1%

## Results

Sampling is taken at a particular location and at a particular instant in time. The sample's water quality results will only relate to what was happening in the river at that location and at that moment in time. Modelling on the other hand simulates both what is happening throughout the river and across the full period of time simulated. The water quality simulations have been carried out over a 15-year period. In order to summarize variations in concentration over a long-term period, the water quality results have been processed into duration exceedance curves. Superimposed on these curves are the constant concentrations which relate to the different classes of water quality for that constituent based on the DOE guidelines.^16^ Given that concentrations vary over time, the question becomes how to relate varying water quality within a river to the class of the river. Conventionally, any class assigned to the river should relate to the class experienced at least 90% of the time. In other words, the assigned class should relate to the class observed (or simulated) at the 10% exceedance probability.

**Figure 1:**
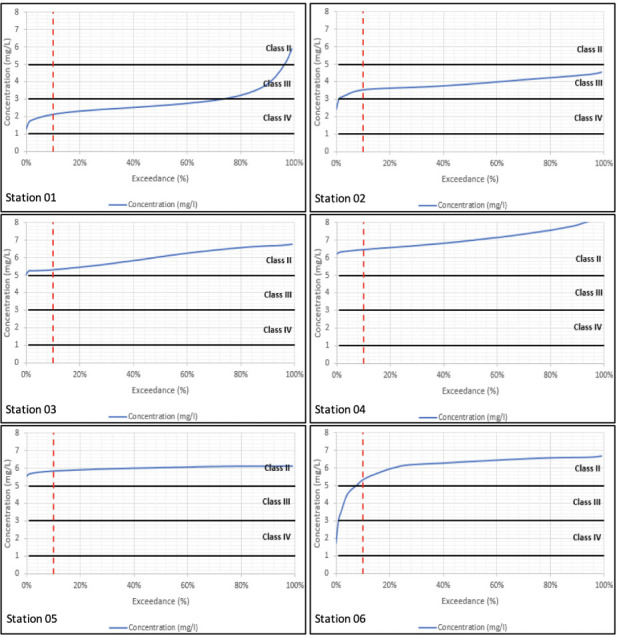
Simulated dissolved oxygen duration exceedance curves at all six sampling stations from downstream (Station 01) to upstream (Station 06)

Water quality duration exceedance curves for long-term model simulations are presented in Figure 1 through Figure 4 with the simulated WQI tabulated in Table 4. Biochemical oxygen demand and NH_4_ parameters fell under Class IV at the majority of the river reaches, while DO varied between Classes II to IV. Total suspended solids within Classes IV to V has been reported, especially along the mid river reaches of the catchment. Comparison across corresponding constituents and locations shows that the water quality model reproduces the long-term duration exceedance for the main body of the curves. However, the water quality model underestimates the infrequent high concentration observations. This is due to the assumption of constant pollutant loads (concentrations) which does not account for all operational practices occurring onsite.

**Figure 2:**
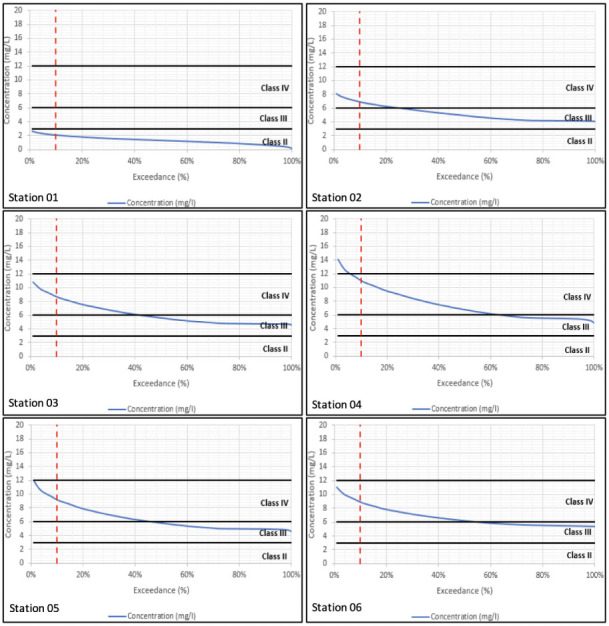
Simulated biochemical oxygen demand duration exceedance curves at all six sampling stations from downstream (Station 01) to upstream (Station 06)

**Figure 3:**
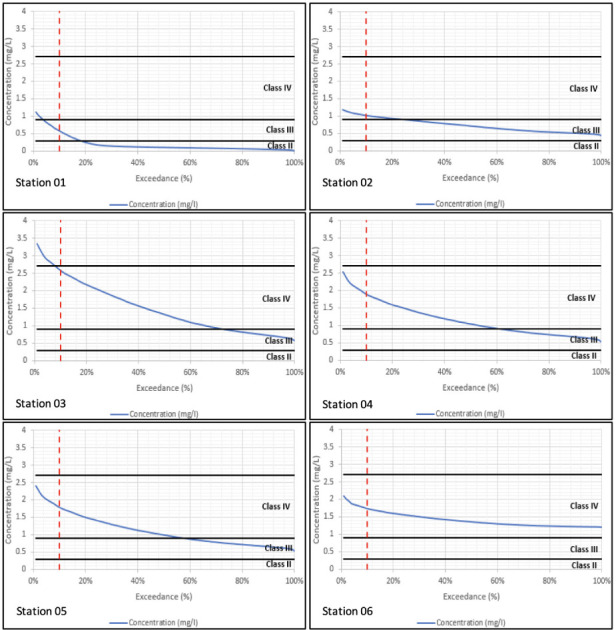
Simulated ammonia duration exceedance curves at all six sampling stations from downstream (Station 01) to upstream (Station 06)

**Figure 4:**
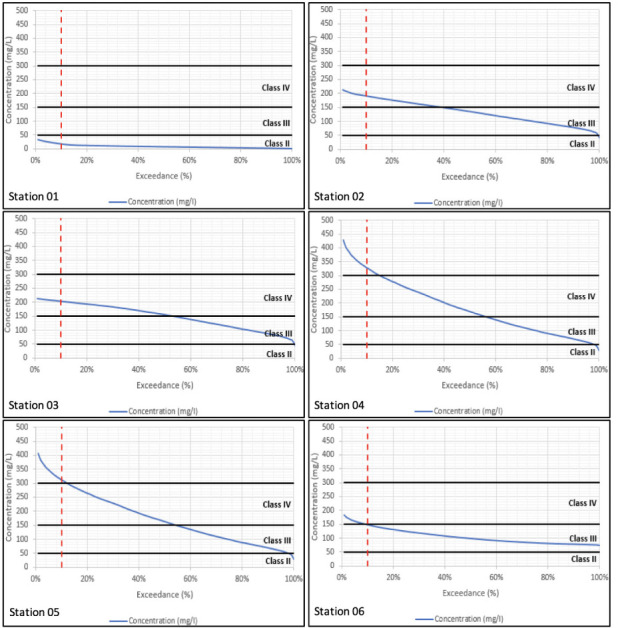
Simulated total suspended solids duration exceedance curves at all six sampling stations from downstream (Station 01) to upstream (Station 06)

**Table 4:** Summary of Simulated Water Quality Index (Baseline)

**Sampling location (downstream-upstream)**	**DO**	**BOD**	**NH_4_**	**TSS**
Station 01 (downstream)	IV	II	III	II
Station 02	III	IV	IV	IV
Station 03	II	IV	IV	IV
Station 04	II	IV	IV	V
Station 05	II	IV	IV	V
Station 06 (upstream)	II	IV	IV	III

In general, the simulated baseline model results show a general consistency of the water quality class compared with the observed data collected at the respective DOE water quality stations. As seen in Figure 1, DO levels generally fall in the downstream direction with the simulated results showing Class IV levels in the intertidal area which is not reflected in the observation. This is likely due to the tidal return parameter that allows too much return flow at the downstream tidal boundary.

In Figure 2, BOD levels were generally high in the upper reaches and improved in the downstream direction as DO is consumed. This is consistent with the reduction in DO in the downstream direction. The results reflect the high BOD load in the upper catchment.

Ammonia levels were generally high in the upper reaches as seen in Figure 3 and improved in the downstream direction. The results mirror the BOD levels and again reflect the high load in the upper catchment.

As seen in Figure 4, TSS levels were very high along most of the river, with reductions at the lower reaches which is more of a reflection of the lower TSS concentration of seawater. The results reflect the high TSS load in the upper catchment of the river.

## Discussion

The water quality modelling results illustrate the connections between nutrient load and site-specific water quality standards of DO, BOD, NH_4_ and TSS. Preliminary analysis of the baseline water quality in the river to date indicates that currently the catchment does not meet the Class II standard. It should be noted that it may not be possible for the catchment to reach Class I standard without extensively improving water quality and reducing the quantity of both point and non-point effluent sources within the catchment. Based on these results, a standard effluent disposal has been proposed to enable the creation of legislation and regulations by authorities in the district that could be replicated for other similar catchments.

In Malaysia, apart from the state of Selangor Waters Management Authority Enactment, national guidelines do not specify numerical limits on discharged pollutant concentrations or loads.^18^ A summary of the pollutant parameters and the standard limit for discharge of pollutant related to aquaculture and sand mining activities in Selangor are outlined in Table 5.

**Table 5:** Summary of Pollutant Parameters and Standard Limit for Discharge of Pollutant Applicable in Selangor (Selangor Regulations)^20^

**Activity**	**Pollutant parameter**	**Standard limit for pollutant discharge**
Fresh water aquaculture in ponds or cage and marine prawn aquaculture in ponds	Ammoniacal nitrogen (NH_3_-N)	5 mg/l
	BOD at 20°C	50 mg/l
	TSS	100 mg/l
	Total nitrogen	10 mg/l
	Total phosphorus	1 mg/l

Mining-related activity	TSS	50 mg/l
	Oil and grease	1 mg/l

The reference Environmental Impact Assessment and Monitoring in Aquaculture presents some numerical limits on discharged pollutant concentrations or loads for aquaculture in various countries, including Australia, India, Japan, Thailand, France, Greece, Poland and Scotland.^19^ We reviewed the above referenced sources and found Selangor Regulations to be slightly more lenient than most international regulations, but in principle it could be used as a reasonable and practical initial guide for specifying numerical limits on discharged pollutant concentrations or loads from aquaculture and sand mining activities.^20^

Adopting the Selangor Regulations with suggested amendments for activities within each individual catchment would be a sensible first step in a proposed roadmap for development of effluent disposal standards for many Malaysian rivers.^20^ It would have the benefit of being consistent with operators in Selangor. The significant drawback to adopting these pollutant concentrations is that they do not quantify the actual pollutant load released to the receiving environment. The load rate can only be quantified by knowing the discharge flow rate and preferably the concentration and flow rate over a suitable time period and at a sensible frequency. Furthermore, it also does not reflect the ambient concentrations (loads) which already exist in the receiving environment, insofar as the carrying capacity of the watercourse may already be exceeded upstream of the discharge.

The main mechanism for controlling discharges from aquaculture activities sits under Section 25 and Section 34A of the Environmental Quality Act (Malaysia).^21^ Many aquaculture farms are smaller than 20 Ha. Unless Section 25 is strictly adhered to, the only practical mechanism at this time is for aquaculture developers and state government to work together outside of legislation towards developing more sustainable aquaculture practices for the preservation of the environment. This can be done by developing best management practice (BMP) guidelines and codes of conduct for voluntary adoption. For example, the Malaysia Department of Fisheries developed a code of conduct for shrimp farming that is an example of general guidelines for voluntary adoption.

In general, the BMPs for sand mining activities include minimizing activities that release fine sediment to the river, retaining vegetation buffers at the edge of waterways and against riverbanks and limiting in-stream operations during the wet season. On the other hand, the BMPs for aquaculture effluent treatment are more diverse and include settlement ponds, filter strips, wetlands, treatment plans, integrated production, injection wells, septic tanks, sterilization/chemical buffering and dilution.

Moving forward, the route that should be taken for many Malaysian rivers is regulatory control by way of the issuance of licenses specifying limits for all effluent discharges from sand mining and aquaculture operations (regardless of operation size). Additionally, these licenses should be issued conditional on certain BMPs being implemented, combined with effluent licenses specifying limits on water quality based on DOE classification as tabulated in Table 2 in conjunction with limited monitoring of operations and discharges.^17^

It should be recognized that using numerical limits for water quality concentrations alone is not fully adequate for environmental protection, since it is the load which is the primary cause of the environmental impact. Furthermore, placing numerical effluent limits on quality or quantity is difficult for discharges which may be highly intermittent (e.g. there are occasional situations like maintenance of a settlement catchment where limits may be exceeded for a short period).

**Figure 5:**
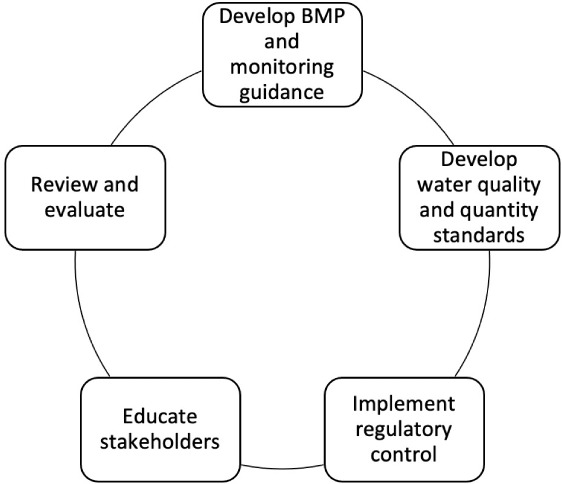
Proposed roadmap for the development of an effluent disposal standard

The BMPs are scalable and can be implemented for operations of any size. Guidance on the use and adoption of BMPs must be an iterative and evolving process to provide a flexible system of pollution abatement so that new approaches can be implemented as technology changes. Best management practices should be selected carefully and adjusted for site-specific conditions due to the diversity of situations and production methods. This proposed roadmap is summarized in Figure 5.

## Conclusions

The present study utilized water quality modelling to investigate the impacts of sand mining and aquaculture management practices in a Malaysian river to inform the connection between the nutrient load and site-specific water quality standards of DO, BOD, NH_4_ and TSS. The model is anticipated to be used in future studies to test various scenarios and mitigation options for informed decision making and evidence-based solutions. The methodological approach showcases the benefits of modelling pollution sources at a local site to describe temporal and spatial variation of physical processes. This is useful for characterizing and analyzing each contributing river branch to inform pollution management strategies. The findings demonstrate the importance of a quantitative effluent disposal standard to manage these impacts in the long term in addition to the best management practice implementation for each activity. Moving forward, a roadmap has been proposed to structure and guide the formulation of a standard effluent disposal guideline for managing these two activities.

## References

[1] Yang B, Lai C, Chen X, Wu X, He Y (2018). Surface water quality evaluation based on a game theory-based cloud model. Water [Internet].

[2] Carre C, Boccarossa A, Hellier E, Dupont N (2018). Quality standards and monitoring as water management tools: the case of nitrogen in rivers in France and Brittany. Water Policy [Internet].

[3] Li S, Chen X, Singh VP, He Y, Bai X (2019). An improved index for water quality evaluation in an estuary region: a case study in the Eastern Pearl River Delta, China. Water Policy [Internet].

[4] McDowell RW, Laurenson S, Van Alfen NK (2014). Water: water quality and challenges from agriculture. Encyclopedia of agriculture and food systems [Internet].

[5] Taylor SD, He Y, Hiscock KM (2016). Modelling the impacts of agricultural management practices on river water quality in Eastern England. J Environ Manag [Internet].

[6] Ott WR (1978). Water quality indices a survey of indices used in the United States [Internet].

[7] Canter LW (1985). Environmental impact of water resources projects [Internet].

[8] Al-Badaii F, Shuhaimi-Othman M, Gasim MB (2013). Water quality assessment of the Semenyih River, Selangor, Malaysia. J Chem [Internet].

[9] Peck Yen T, Rohasliney H (2013). Status of water quality subject to sand mining in the Kelantan river, Kelantan. Trop Life Sci Res.

[10] Hashim N, Teo FY Water quality modelling of Kuyoh River basin Malaysia.

[11] Othman F, Elamin AE, Azahar SA, Muhammad SA Utilizing GIS in the development of water quality river modeling for Penchala River, Malaysia. Appl Mech Mater.

[12] Othman F, Elamin AE (c2014). Linking the water quality model with GIS and ambient phenomena.

[13] Aminu M, Matori AN, Yusof KW, Malakahmad A, Zainol RB (2015). A GIS-based water quality model for sustainable tourism planning of Bertam River in Cameron Highlands, Malaysia. Environ Earth Sci [Internet].

[14] Liang J, Yang Q, Sun T, Martin JD, Sun H, Li L (2015). MIKE 11 model-based water quality model as a tool for the evaluation of water quality management plans. J Water Supply [Internet].

[15] Hutchins MG, Bowes M (2018). Balancing water demand needs with protection of river water quality by minimising stream residence time: an example from the Thames, UK. Water Resour Manag [Internet].

[16] Danish Hydraulic Institute (2017). MIKE ECO Lab - Numerical Lab for Ecological and Agent Based Modelling User Guide. https://manuals.mikepoweredbydhi.help/2017/Coast_and_Sea/ABM_StepbyStep_1_DrifterExample.pdf.

[17] (2009). Malaysia environmental quality report.

[18] **Selangor Waters Management Authority (Malaysia)** Enactment No. 2 of 1999, Selangor Waters Management Authority Enactment 1999 Arrangement of Sections: Part I Preliminary Section

[19] (2009). Environmental impact assessment and monitoring in aquaculture: requirements, practices, effectiveness and improvements [Internet]. http://www.fao.org/3/i0970e/i0970e.pdf.

[20] **Selangor Waters Management Authority (Malaysia)** Enactment 1999, Emission or Discharge of Pollutants (State of Selangor) Regulations 2012, Arrangement of Regulations: Part 1 Preliminary

[21] Laws of Malaysia, Reprint. Act 127 Environmental Quality Act 1974: Incorporating all amendments up to 1 January 2006

